# Antimicrobial use in European long-term care facilities: results from the third point prevalence survey of healthcare-associated infections and antimicrobial use, 2016 to 2017

**DOI:** 10.2807/1560-7917.ES.2018.23.46.1800394

**Published:** 2018-11-15

**Authors:** Enrico Ricchizzi, Katrien Latour, Tommi Kärki, Rossella Buttazzi, Béatrice Jans, Maria Luisa Moro, Olivia Aya Nakitanda, Diamantis Plachouras, Dominique L Monnet, Carl Suetens, Pete Kinross, the HALT Study Group

**Affiliations:** 1 Agenzia sanitaria e sociale regionale – Regione Emilia Romagna, Bologna, Italy; 2 Sciensano, Brussels, Belgium; 3 European Centre for Disease Prevention and Control, Solna, Sweden; 4 Members of the HALT Study Group have been listed at the end of this article

**Keywords:** antimicrobial use, long-term care facility, LTCF, antimicrobial resistance, AMR, point-prevalence survey, PPS, surveillance, healthcare-associated infections, HAI

## Abstract

Antimicrobials are commonly prescribed and contribute to the development of antimicrobial resistance in long-term care facilities (LTCFs). In 2010, the European Centre for Disease Prevention and Control initiated point prevalence surveys (PPS) of healthcare-associated infections and antimicrobial use in European LTCFs, performed by external contractors as the Healthcare-Associated infections in Long-Term care facilities (HALT) projects. Here, we investigated prevalence and characteristics of antimicrobial use and antimicrobial stewardship indicators in European LTCFs in 2016–17. Twenty-four European Union/European Economic Area (EU/EEA) countries, the former Yugoslav Republic of Macedonia and Serbia participated in the third PPS in European LTCFs. Overall, 4.9% (95% confidence interval: 4.8–5.1) of LTCF residents in the EU/EEA participating countries received at least one antimicrobial. The most commonly reported Anatomical Therapeutic Chemical (ATC) groups were beta-lactam antibacterials/penicillins (J01C), other antibacterials (J01X) (e.g. glycopeptide antibacterials, polymyxins), quinolones (J01M), sulfonamides and trimethoprim (J01E), and other beta-lactams (J01D). Urinary tract infections and respiratory tract infections were the main indications for antimicrobial prescription. This PPS provides updated and detailed information on antimicrobial use in LTCFs across the EU/EEA that can be used to identify targets for future interventions, follow-up of these interventions and promote prudent use of antimicrobials in European LTCFs.

## Introduction

Life expectancy is increasing steadily in the European Union/European Economic Area (EU/EEA). Population projections estimate that by 2050 the old-age dependency ratio, calculated as the number of individuals aged over 65 years per 100 people of working age, will reach 50% [1]. The ageing population is one reason for the transitions in healthcare delivery systems taking place in several EU/EEA countries. This includes reductions in hospital beds and in several countries more patient care being provided in long-term care settings [2]. Long-term care facilities (LTCFs) deliver a blend of health and social services to people who are limited in their ability to live independently, especially due to old age, and are in need of less intensive medical care than that usually provided in hospitals [3].

Despite the fact that less intensive medical care is provided in LTCFs than in hospitals, healthcare-associated infections (HAIs) are common in the vulnerable LTCF populations [4-9]. For this reason, antimicrobials are commonly prescribed in LTCFs, contributing to the development of antimicrobial resistance (AMR) and possibly leading to adverse events such as *Clostridium difficile* infection, and infections that are more difficult to treat [10,11]. As there is increasing evidence that LTCFs can serve as a reservoir for the transmission of resistant organisms to other healthcare settings, close monitoring of the situation is needed [12,13]. Furthermore, the lack of diagnostic capabilities may lead to suboptimal antimicrobial prescription in LTCFs [14,15].

Data on antimicrobial use in LTCFs are necessary to understand the reasons, magnitude and determinants of antimicrobial prescribing and to inform public health policies on prudent use of antimicrobials. In June 2017, the European Commission published guidelines for the prudent use of antimicrobials in human medicine, recommending to establish antimicrobial stewardship programmes in all healthcare facilities, including LTCFs [16]. Although several European countries already measure antimicrobial consumption, methodologies have not been consistent precluding meaningful comparisons, furthermore they have often concentrated in the acute care settings, with little attention given to LTCFs.

For this reason, the European Centre for Disease Prevention and Control (ECDC) initiated surveillance of HAIs and antimicrobial use in European LTCFs with point prevalence surveys (PPSs) under the Healthcare-Associated infections in Long-Term Care facilities (HALT) projects in 2010, 2013 and, most recently, in 2016–17. In the present study, we investigated the prevalence and characteristics of antimicrobial use and antimicrobial stewardship indicators in European LTCFs reported in the third European PPS of HAIs and antimicrobial use in LTCFs (HALT-3) in 2016–17.

## Methods

### Survey design

The survey was performed in 24 EU/EEA countries and two EU candidate countries, the former Yugoslav Republic of Macedonia and Serbia. The countries were asked to recruit LTCFs in their country for participation in the survey. According to the protocol [17], the selected LTCFs had to provide a broad range of services and assistance to people with limited abilities to function independently on a daily basis (i.e. to autonomously perform the basic activities of daily living over an extended period of time). In addition, these LTCFs could also provide basic medical services (wound dressing, pain management, medication, health monitoring, prevention, rehabilitation or palliative care), but the LTCF residents had to be medically stable, without the need for constant specialised medical care or invasive medical procedures. Resident stay in the selected LTCFs could vary from temporary to permanent (until end of life).

To improve country representativeness, a recommended minimum number of LTCFs per country was calculated and provided to the national coordinators. For each country, the recommended sample size was calculated anticipating a national crude HAI prevalence of 4%, with a 95% confidence interval (CI) of 3‒5% (1% precision). Although representative sampling was strongly recommended, purposive sampling, including convenience sampling or voluntary participation after the invitation of all LTCFs, was also accepted. Different types of LTCF could be recruited. While also specialised LTCF types (such as psychiatric facilities, rehabilitation centres and palliative care centres) were invited to participate, only data from general nursing homes (providing principally care to seniors with severe illnesses or injuries), residential homes (facilities usually providing personal care, housekeeping and three meals a day) and mixed LTCFs (providing mixed services for elderly or other resident populations) were considered for analysis. For countries contributing to the survey with more residents than in the calculated recommended sample size, a randomised sub-sample was used in the final analysis [17].

### Data collection

Participating countries were asked to organise the survey during one of four proposed periods: April–June or September–November in 2016 or 2017. Ideally, data had to be collected on a single day for each LTCF. In large LTCFs, data collection could take place over 2 or more consecutive days, but all residents within one ward or unit had to be surveyed on the same day.

Data collection was conducted either by an external data collector (i.e. the national coordinator or a person trained by the national coordinator) or by a local data collector (i.e. an LTCF staff member, e.g. designated physician, infection control practitioner or nurse). To ensure standardisation of data collection, a ‘train-the-trainers’ workshop for the national coordinators was held in December 2015. It was recommended that national coordinators organise at least one 1-day information and training session for the LTCFs before the national survey [17].

A resident questionnaire was used to collect data for each resident receiving a systemic antimicrobial on the day of the survey. Data included resident characteristics (age, gender, length of stay in the LTCF (less or greater than 1 year)), risk factors (urinary catheter, vascular catheter, pressure sores, other wounds), care load indicators (faecal and/or urinary incontinence, disorientation in time and/or space, impaired mobility) and antimicrobial use (name of antimicrobial agent(s), indication and reasons for antimicrobial use, place of prescription, administration route, end or review date of documented prophylaxis or treatment) [17].

The 2018 version of the Anatomical Therapeutic Chemical/Defined Daily Dose (ATC/DDD) Index of the World Health Organization Collaborating Centre for Drug Statistics Methodology was used to classify the antimicrobials into different groups [18]. Antimicrobial agents for systemic use within ATC groups A07AA (intestinal antiinfectives), D01BA (dermatological antifungals for systemic use), J01 (antibacterials for systemic use), J02 (antimycotics for systemic use), J04 (antimycobacterials), when used for treatment of mycobacteria (including tuberculosis) or as reserve for multidrug-resistant bacteria and P01AB (nitroimidazole-derived antiprotozoals), were included. Antiviral agents were not included.

Two main indications for antimicrobial use were recorded, i.e. prophylaxis and treatment. The indication was further divided according to the anatomical site or diagnosis of prophylaxis or treatment: urinary tract, genital tract, skin or wound, respiratory tract, gastrointestinal tract, eye, ear-nose-mouth, surgical site, tuberculosis, systemic infection, unexplained fever or other site or diagnosis not previously specified.

An LTCF institutional questionnaire was used to collect data on structures and processes in place in each participating LTCF, including current infection control practices and antimicrobial policies, e.g. written guidelines for appropriate antimicrobial use in the facility, annual regular training on appropriate antimicrobial prescribing or a ‘restrictive list’ of antimicrobials to be prescribed. In addition, anonymised and aggregated denominator data were also collected for the entire eligible LTCF population and included information on gender distribution, as well as the proportion of residents aged over 85 years who were receiving at least one antimicrobial agent, were disoriented in time and/or space, had urinary and/or faecal incontinence, had impaired mobility, had pressure sores, had a urinary catheter, had a vascular catheter, had other wounds and/or had surgery in the previous 30 days.

### Statistical analysis

All data were checked for errors, omissions and inconsistent answers on the national level and centrally before analysis.

Analyses were performed in SAS 9.3 (SAS Institute, Cary, NC, United States) and R 3.5.0 (R Foundation for Statistical Computing, Vienna, Austria). We calculated the crude, pooled prevalence of antimicrobial use as the number of residents receiving at least one antimicrobial agent divided by the total number of eligible residents on the day of the survey. We also calculated the mean, median and interquartile range (IQR) for the prevalence of antimicrobial use for the included LTCFs overall and within each country.

Multivariable linear regression was used to assess the association between antimicrobial use on the day of the survey and the type and size of LTCFs, as well as characteristics of the LTCF resident population, including care load indicators. Countries reporting data by LTCF ward without indication of the corresponding LTCF (Portugal and Sweden), or data from LTCFs with missing population data on the LTCF questionnaire (France and Norway), as well as LTCFs which reported a prevalence of antimicrobial use of more than 60%, were excluded from this analysis. The latter were considered outliers and represented less than 0.2% of all participating LTCFs.

### Ethical considerations and confidentiality

Each participating country had different requirements for ethical approval for the survey, with some requiring approval from an ethics committee as well as written informed consent of the residents (or their proxies). Confidentiality of the data was ensured by the use of a unique, coded survey identification number for each LTCF and for each resident.

## Results

### Participation

In total, 3,052 LTCFs with 181,462 eligible residents from 24 EU/EEA countries participated in the survey. After adjustment for over-representation of countries contributing to the survey with more than the recommended number of residents, 102,301 eligible residents from 1,788 LTCFs remained in the dataset used for this analysis (Table 1). Data from the United Kingdom (UK) were reported separately for three administrations: UK-Northern Ireland, UK-Scotland and UK-Wales. UK-England did not participate in the survey. The Czech Republic only provided institutional-level data for nine LTCFs and was therefore excluded in the antimicrobial use and resident data analysis.

**Table 1:** Prevalence of antimicrobial use, by country, 23 European Union/European Economic Area countries^a^, the former Yugoslav Republic of Macedonia and Serbia, 2016–2017

Country	LTCFs	Eligible residents	Antimicrobial use			
			Residents with at least one antimicrobial	Observed prevalence	Mean prevalence of LTCFs	Median prevalence of LTCFs
	n	n	n	% (95% CI)	%	IQR (%)
Austria	12	2,065	67	3.2 (2.5 to 4.1)	2.9	2.4 (1.0 to 4.7)
Belgium	79	8,206	482	5.9 (5.4 to 6.4)	5.8	5.1 (2.9 to 8.1)
Croatia	8	1,607	32	2.0 (1.4 to 2.8)	3.2	3.6 (0.8 to 4.9)
Cyprus	11	312	29	9.3 (6.3 to 13.1)	10.1	7.7 (4.8 to 17.0)
Denmark	95	3,346	350	10.5 (9.4 to 11.5)	10.7	9.0 (6.3 to 15.0)
Finland	149	5,914	394	6.7 (6.0 to 7.3)	7.0	5.9 (2.3 to 10.5)
France	91	6,957	187	2.7 (2.3 to 3.1)	2.7	2.3 (0 to 4.3)
Germany	82	6,705	85	1.3 (1.0 to 1.6)	1.3	0.9 (0 to 1.9)
Greece	13	812	49	6.0 (4.5 to 7.9)	7.5	4.2 (3.0 to 11.6)
Hungary	75	7,670	71	0.9 (0.7 to 1.2)	0.9	0 (0 to 1.4)
Ireland	109	5,613	543	9.7 (8.9 to 10.5)	11.7	8.6 (5.4 to 14.7)
Italy	196	11,417	495	4.3 (4.0 to 4.7)	5.5	3.1 (0.8 to 6.6)
Lithuania	26	3,438	25	0.7 (0.5 to 1.1)	0.9	0 (0 to 1.0)
Luxembourg	16	1,616	42	2.6 (1.9 to 3.5)	2.5	1.5 (0.9 to 4.2)
Malta	11	2,485	66	2.7 (2.1 to 3.4)	1.6	1.4 (0.5 to 2.4)
The Netherlands	57	4,547	202	4.4 (3.9 to 5.1)	5.1	4.3 (1.6 to 6.7)
Norway	62	2,447	169	6.9 (5.9 to 8.0)	7.0	4.6 (2.1 to 10.3)
Poland	24	2,281	73	3.2 (2.5 to 4.0)	4.4	2.9 (0.9 to 6.5)
Portugal	132	3,633	220	6.1 (5.3 to 6.9)	6.8	4.3 (0 to 10.0)
Slovakia	59	5,091	113	2.2 (1.8 to 2.7)	2.9	1.2 (0 to 3.4)
Spain	46	6,808	717	10.5 (9.8 to 11.3)	11.7	10.8 (3.5 to 17.3)
Sweden	285	3,604	118	3.3 (2.7 to 3.9)	3.2	0 (0 to 5.6)
UK – Northern Ireland	70	2,614	270	10.3 (9.2 to 11.6)	10.4	9.8 (5.0 to 14.3)
UK – Scotland	52	2,147	138	6.4 (5.4 to 7.5)	6.2	5.1 (0 to 10.9)
UK – Wales	28	966	98	10.1 (8.3 to 12.2)	10.1	8.2 (5.5 to 11.4)
**EU/EEA**	**1,788**	**102,301**	**5,035**	**4.9 (4.8 to 5.1)**	**5.8**	**3.6 (0 to 8.5)**
former Yugoslav Republic of Macedonia	4	294	26	8.8 (5.9 to 12.7)	5.2	5.1 (2.5 to 7.9)
Serbia	6	1,168	57	4.9 (3.7 to 6.3)	6.0	4.0 (3.7 to 5.5)

CI: confidence interval; EU/EEA: European Union/European Economic Area; IQR: interquartile range; LTCFs: long-term care facilities; UK: United Kingdom.

^a^For the United Kingdom, data for Northern Ireland, Scotland and Wales are presented separately. England did not participate in the survey. The Czech Republic did not provide resident-level data.

### Antimicrobial use and resident data

On the day of the survey, 5,035 residents received at least one antimicrobial agent, resulting in a crude, pooled prevalence of antimicrobial use of 4.9% (95% CI: 4.8 to 5.1). The mean antimicrobial use prevalence of LTCFs was 5.8% and the median was 3.6% (interquartile range (IQR): 0.0–8.5) (Table 1).

Detailed information on antimicrobial prescribing was provided for 5,006 residents (i.e. all participating countries except Cyprus and the Czech Republic). The median age of residents was 85 years; 65.7% were female and 93.8% received one antimicrobial agent, while 5.8% received two and 0.4% received more than two. In total, 5,344 antimicrobial agents were reported to have been given on the day of the survey, an average of 1.07 antimicrobial agents per resident. Antimicrobials were mainly administered orally (88.1%) The parenteral route (intramuscular or intravenous) was used for 10.9% of prescribed antimicrobials and nasal or rectal administration route was reported for only 0.7% of prescribed antimicrobials.

**Figure 1:**
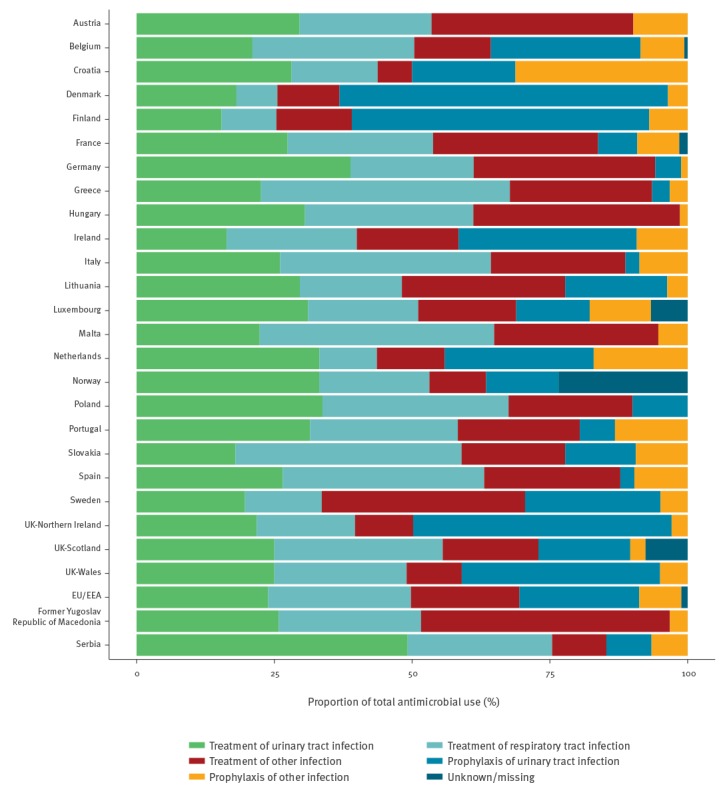
Indications (treatment or prophylaxis, for the most commonly sites of infection) for antimicrobial use in long-term care facilities, by country, 22 European Union/European Economic Area countries^a^, the former Yugoslav Republic of Macedonia and Serbia, 2016–2017

Antimicrobials were most frequently prescribed within the same LTCF (77.9%), followed by an acute care hospital (12.9%) or another location (5.1%), with no data provided for the remaining 4.2%. The indication was reported as treatment for 69.5% and prophylaxis for 29.4% of prescribed antimicrobials, and indication was missing for the remaining 1.1%. An end or review date for the prescription was documented for 64.6% of prescribed antimicrobials and was higher for treatment (81.6%) than for prophylaxis (26.2%). Figure 1 shows the distribution of antimicrobial use by indication and common site of infection for the EU/EEA overall and for each country.

EU/EEA: European Union/European Economic Area.

^a^For the United Kingdom, data for Northern Ireland, Scotland and Wales are presented separately. England did not participate in the survey. The Czech Republic did not provide resident-level data. Cyprus did not provide detailed information on antimicrobial prescribing.

Overall, the urinary tract was the most common body site for which antimicrobials were prescribed (46.1%), followed by respiratory tract (29.4%) and skin or wound (12.6%). Combined, these sites accounted for 88.0% of all antimicrobial prescriptions. When stratified by indication, the most common sites for antimicrobial treatment were the respiratory tract (37.2%), urinary tract (34.4%), skin or wound (15.8%) and gastrointestinal tract (2.8%). For prophylaxis, the urinary tract was the most common body site (74.0%), followed by respiratory tract (11.3%), skin or wound (4.8%), another non-specified body site (3.4%) and gastrointestinal tract (2.4%).

Antibacterials for systemic use (ATC J01) accounted for 95.4% of all antimicrobial prescriptions. Other antimicrobial groups accounted for the remaining 4.6%, i.e. nitroimidazole derivatives (P01AB, 1.5%), intestinal anti-infectives–antibiotics (A07AA, 1.3%), antimycotics for systemic use (J02, 1.2%), antimycobacterials for treatment of tuberculosis (J04A, 0.5%) and antifungals for systemic use (D01B, 0.2%).

**Figure 2:**
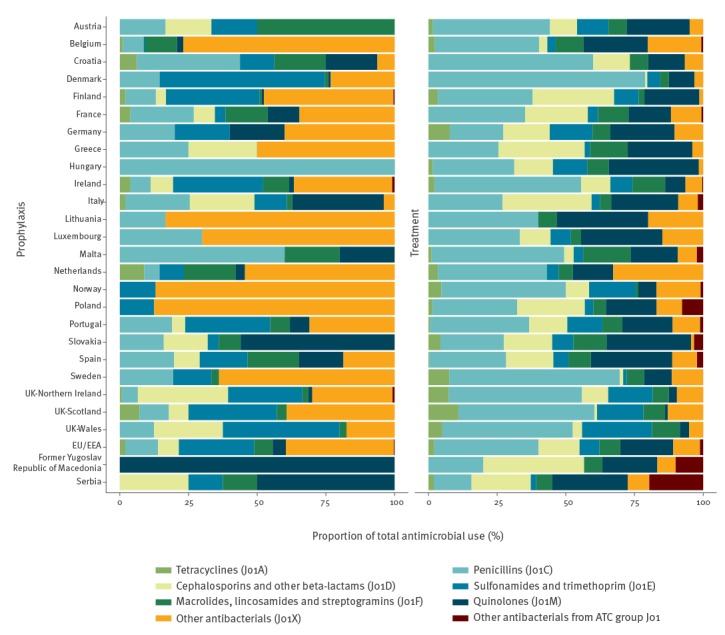
Distribution of antibacterials for systemic use (ATC group J01) into groups, by main indication (prophylaxis or treatment) and by country, 22 European Union/European Economic Area countries^a^, the former Yugoslav Republic of Macedonia and Serbia, 2016–2017

In total, 5,098 prescriptions of antibacterials for systemic use (ATC J01) were reported. Within this group, the most frequently reported subgroups were: beta-lactam antibacterials, penicillins (J01C: 30.2%), other antibacterials (J01X: 18.6%), quinolones (J01M: 14.9%), sulfonamides and trimethoprim (J01E: 13.3%) and other beta-lactams (J01D: 12.6%). Other groups accounted for the remaining 10.4% of antibacterials for systemic use. Figure 2 shows the distribution of antibacterials for systemic use by indication (prophylaxis or treatment) and by country.

EU/EEA: European Union/European Economic Area.

^a^For the United Kingdom, data for Northern Ireland, Scotland and Wales were reported separately. England did not participate in the survey. The Czech Republic did not provide resident-level data. Cyprus did not provide detailed information on antimicrobial prescribing.

For prophylaxis of urinary tract infection (UTI), the most frequently used antimicrobial agents were trimethoprim (J01EA01: 29.7%), nitrofurantoin (J01XE01: 27.0%), methenamine (J01XX05: 11.6%), cefalexin (J01DB01: 6.1%) and fosfomycin (J01XX01: 5.9%); these accounted for 81.8% of all antimicrobials used for prophylaxis of UTI.

The LTCF and LTCF population characteristics associated with prevalence of antimicrobial use, as identified in the multivariable linear regression analysis, are presented in Table 2. The regression model indicated that LTCF and LTCF population characteristics only explained 19% of the variance in the prevalence of antimicrobial use (R^2^ = 0.1889). Prevalence of antimicrobial use was significantly higher in mixed LTCFs, as well as in LTCFs with less than 65 beds. For the demographic characteristics, for one percent increase in the proportion of male residents the prevalence of antimicrobial use increased by 7%. For one percent increase in the proportion of residents over 85 years of age, the prevalence of antimicrobial use increased by 5%. For the care load indicators and risk factors, the most significant increases in antimicrobial use prevalence were associated with the proportion of residents with a vascular catheter and with surgery in the previous 30 days; for one percent increase in the proportion of these risk factors, the prevalence increased by 26% and 20%, respectively.

**Table 2:** Multivariable linear regression analysis of long-term care facility and resident characteristics in relation to the prevalence of antimicrobial use, 19 European Union/European Economic Area countries^a^, the former Yugoslav Republic of Macedonia and Serbia, 2016–2017

Characteristics	Coefficient (95% CI)	p-value
**Type of LTCF**		
Residential home	Ref	
General nursing home	0.38 (-0.54 to 1.31)	0.418
Mixed	1.41 (0.40 to 2.42)	**0.006**
**Size of LTCF**		
≥ 105 beds	Ref	
65–104 beds	0.62 (-0.47 to 1.71)	0.266
37–64 beds	2.25 (1.22 to 3.29)	**< 0.001**
< 37 beds	3.27 (2.25 to 4.29)	**< 0.001**
**Characteristics of LTCF residents (%)**		
Aged over 85 years	0.05 (0.03 to 0.08)	**< 0.001**
Male	0.08 (0.05 to 0.11)	**< 0.001**
Using a wheelchair or bedridden	-0.04 (-0.06 to -0.02)	**< 0.001**
Disoriented in time and/or space	0.00 (-0.01 to 0.02)	0.648
Urinary and/or faecal incontinence	0.02 (-0.00 to 0.04)	0.052
Pressure sore	-0.03 (-0.09 to 0.02)	0.229
Other wound	0.10 (0.06 to 0.14)	**< 0.001**
Surgery in the previous 30 days	0.20 (0.10 to 0.30)	**< 0.001**
Urinary catheter	0.04 (0.00 to 0.08)	**0.043**
Vascular catheter	0.26 (0.18 to 0.33)	**< 0.001**

CI: confidence interval; EU/EEA: European Union/European Economic Area; LTCF: long-term care facility.

^a^For the United Kingdom, data for Northern Ireland, Scotland and Wales were reported separately. England did not participate in the survey. The Czech Republic did not provide resident-level data. France, Portugal, Norway and Sweden were excluded from the multivariable analysis (see Methods).

Significant p-values are shown in bold.

### Antimicrobial stewardship indicators

Of the antimicrobial stewardship indicators reported at LTCF level, the most common was ‘written guidelines for appropriate antimicrobial use in the LTCF’ (39.4%). Annual regular training on appropriate antimicrobial prescribing was reported by 20.7% of LTCFs included in the sample. Having a ‘restrictive list’ of antimicrobials was reported by 24.0% of LTCFs; the antimicrobials most commonly restricted were carbapenems (J01DH, 70.1%), parenteral vancomycin (J01XA01, 63.7%), all intravenously administered antibiotics (53.9%), glycopeptides (J01XA, 53.9%), third-generation cephalosporins (J01DD, 45.3%), ‘broad-spectrum antibiotics’ (41.9%), fluoroquinolones (J01MA, 32.8%) and mupirocin (D06AX09 and R01AX06, 21.3%) (Table 3).

**Table 3:** Structure and process indicators of antimicrobial stewardship reported in participating LTCFs, by country, 23 European Union/European Economic Area countries^a^, the former Yugoslav Republic of Macedonia and Serbia, 2016–2017

Country^a^	Responding LTCFs	Written guidelines for appropriate antimicrobial use in the LTCF		Annual regular training on appropriate antimicrobial prescribing		Responding LTCFs	A ‘restrictive list’ of antimicrobials to be prescribed		Antimicrobial groups reported as being restricted (‘restrictive list’)(ATC code)							
									J01DD	J01MA	J01DH	J01XA	J01XA01	IAA	BSA	D06AX09, R01AX06
	n	n	%	n	%	n	n	%	n	n	n	n	n	n	n	n
Austria	12	9	75.0	2	16.7	12	2	16.7	0	0	2	1	0	0	0	0
Belgium	78	27	34.6	5	6.4	79	11	13.9	1	1	2	2	2	3	2	5
Croatia	8	1	12.5	0	0	8	1	12.5	1	0	0	0	0	0	1	0
Cyprus	11	2	18.2	1	9.1	11	1	9.1	1	1	0	0	1	0	0	0
Czech Republic	9	1	11.1	1	11.1	9	1	11.1	0	0	1	1	1	0	0	0
Denmark	95	2	2.1	0	0	95	1	1.1	0	0	0	0	0	0	0	0
Finland	147	20	13.6	7	4.8	149	4	2.7	0	0	0	0	0	4	0	0
Germany	82	1	1.2	2	2.4	82	0	0.0	0	0	0	0	0	0	0	0
Greece	13	0	0	0	0	13	5	38.5	4	4	4	4	4	4	4	4
Hungary	72	6	8.3	2	2.8	75	10	13.3	0	0	2	2	5	10	1	0
Ireland	106	41	38.7	8	7.5	109	15	13.8	6	1	7	2	5	6	1	3
Italy	193	41	21.2	19	9.8	195	110	56.4	36	19	91	60	77	45	29	16
Lithuania	26	0	0	0	0	26	1	3.8	0	0	1	1	1	0	0	1
Luxembourg	16	1	6.3	0	0	16	0	0.0	0	0	0	0	0	0	0	0
Malta	11	5	45.5	1	9.1	11	0	0.0	0	0	0	0	0	0	0	0
The Netherlands^b^	21	21	100	NA^c^	NA	22	21	95.5	0	0	0	0	0	0	0	0
Norway	51	39	76.5	9	17.6	NA^c^	NA	NA	NA	NA	NA	NA	NA	NA	NA	NA
Poland	24	7	29.2	2	8.3	24	8	33.3	4	2	6	4	5	2	3	0
Portugal	130	49	37.7	28	21.5	132	102	77.3	51	35	67	53	68	57	48	48
Slovakia	59	19	32.2	0	0	59	59	100.0	59	59	59	59	59	59	59	0
Spain	42	31	73.8	14	33.3	46	25	54.3	6	0	21	13	11	5	7	3
Sweden	285	285	100	236	82.8	285	0	0.0	0	0	0	0	0	0	0	0
UK – Northern Ireland	70	20	28.6	2	2.9	70	2	2.9	0	0	0	0	0	2	0	0
UK –Scotland	52	15	28.8	1	1.9	51	5	9.8	1	1	0	0	0	4	1	0
UK – Wales	26	3	11.5	0	0	28	2	7.1	0	0	0	0	0	1	1	0
**EU/EEA**	**1 639**	**646**	**39.4**	**340**	**20.7**	**1 607**	**386**	**24.0**	**170**	**123**	**263**	**202**	**239**	**202**	**157**	**80**
Former Yugoslav Republic of Macedonia	4	1	25.0	1	25.0	4	0	0.0	0	0	0	0	0	0	0	0
Serbia	6	2	33.3	1	16.7	6	1	16.7	1	1	0	0	0	1	1	0

BSA: Broad-spectrum antibiotics; D06AX09, R01AXA6: Mupirocin; EU/EAA: European Union/European Economic Area; IAA: Intravenously-administered antibiotics; J01DD: Third-generation cephalosporins; J01DH: Carbapenems; J01MA: Fluoroquinolones; J01XA: Glycopeptides; J01XA01: Vancomycin (parenteral); LTCF: long-term care facility; NA: not available.

^a^For the United Kingdom, data for Northern Ireland, Scotland and Wales are presented separately. England did not participate in the survey.

^b^Only a limited number of participating LTCFs in the Netherlands collected antimicrobial stewardship data.

^c^Data were not collected.

France did not provide data for the items presented in the table.

## Discussion

This study examined antimicrobial prescribing in LTCFs in 24 EU/EEA countries. The crude prevalence of residents receiving at least one antimicrobial agent was 4.9%; the majority of antimicrobials being administered orally. Antimicrobials were more frequently prescribed for the treatment of an infection, while almost one third were given as prophylaxis. The crude prevalence of antimicrobial use in this survey in 2016–17 was similar to that reported in previous similar HALT surveys from 2010 (4.3%) and 2013 (4.4%) [19,20]. UTIs and respiratory tract infections were the main indications for antimicrobial use, both for treatment or as prophylaxis. This and previous similar surveys in the EU/EEA consistently show large variations of antimicrobial prescribing practices in LTCFs, across and within participating countries [19-21]. The prevalence of residents receiving antimicrobials for prophylaxis also varied largely across countries. In Denmark and Finland, prophylaxis was reported more frequently than treatment, confirming the high proportion of prophylaxis reported in previous surveys from these countries [19,20].

The most commonly prescribed antimicrobials were: penicillins, other antibacterials, quinolones, sulfonamides and trimethoprim, and other beta-lactams. Penicillins, other antibacterials and quinolones were also the most frequently prescribed antimicrobials in both the 2010 and 2013 HALT surveys. For UTI prophylaxis, other antibacterials, sulfonamides and trimethoprim, and penicillins were the most commonly prescribed antimicrobials, as in both the 2010 and 2013 surveys [19,20].

There is variation within the EU/EEA in what is considered long-term care with regard to sheltered housing, length of stay and range of beneficiaries, as well as an absence of a clear division between medical and social services [22]. To enhance comparability, we only included nursing homes, residential homes and mixed LTCFs in this analysis. Despite this, we noted differences in the case-mix of resident populations. For example, Spain reported that post-acute care residents were commonly included to the surveyed population. In the Netherlands, the level of care provided in the LTCFs covers residents that previously would have often been admitted to a hospital. Therefore, such differences in the definition of long-term care might partially explain a high prevalence of antimicrobial use in some EU/EEA countries. The large variation between LTCFs in the prevalence of residents with a vascular catheter or with previous surgery is an indication that some of the participating LTCFs could, in fact, be step-down facilities with a very different resident case-mix than an average nursing home.

Large differences were observed in the prevalence of care load indicators and risk factors between countries, as well as within each country (unpublished data). Our multivariable analysis showed that several of these indicators and risk factors were independently and positively associated with prevalence of antimicrobial use. However, our model that took into account LTCF characteristics and resident characteristics, including care load and risk factors, only explained 19% of the variation in the prevalence of antimicrobial use in LTCFs in EU/EEA countries. This suggests that other factors, such as national or regional regulations on antimicrobial use, as well as local habits and prescriber preferences and practices, have a larger impact than characteristics of the residents’ population [23]. In this survey, prophylaxis of UTI was a frequent indication for antimicrobial use in LTCFs, remaining the most common indication in several countries and showing no significant decline since the HALT surveys performed in 2010 or 2013 [19,20]. Although evidence suggests that long-term antimicrobials for prophylaxis may reduce the risk of recurrence of UTIs in women [24], this benefit diminishes immediately on cessation of antimicrobial use and, more importantly, is associated with a large increase in the proportion of antibiotic-resistant bacteria isolated from urine and faeces. Therefore, the practice of prescribing antimicrobials for prophylaxis of UTI should be carefully evaluated, and more studies about the effectiveness of prophylaxis of UTIs in the LTCF populations may be needed, depending also on the chosen antimicrobial. For example, the characteristics of methenamine (ATC J01XX05) are very different from that of other antimicrobials commonly prescribed for prophylaxis of UTI [25,26].

Information on antimicrobial stewardship indicators was collected to describe the resources available in LTCFs to support rational use of antimicrobials. Documentation of the end or review date for the prescription in the residents’ notes is an indicator of the quality of antimicrobial prescription, and this end or review date was documented for almost two out of three prescriptions overall; however, end or review dates were only reported in one out of four prescriptions for prophylaxis. Other antimicrobial stewardship indicators, such as guidelines for appropriate use, were reported by a small proportion of LTCFs in the EU/EEA. Some countries, such as France, Germany, the Netherlands and Norway, reported the dissemination of national guidelines and Norway and the Netherlands reported that the guidelines were specific for the elderly patient population. The antimicrobial stewardship indicator data in this survey were comparable with that from previous similar surveys, which indicate that improvements in antimicrobial stewardship are urgently needed in LTCFs in the EU/EEA [16,27].

The strengths of this survey include the use of a standardised protocol across all participating LTCFs, the collection of detailed data on the LTCF characteristics and antimicrobial stewardship practices and the inclusion of a wide variety of LTCF residents and data on their antimicrobial use. The survey is characterised by broad participation and a very large sample size, providing a good overall picture of antimicrobial use in LTCFs in the EU/EEA, with meaningful benchmarks for participating countries and LTCFs. Considering the participation and representativeness of the current survey, it is important to note that the overall number of participating countries increased from the previous HALT survey in 2013; in addition, the number of participating LTCFs increased progressively between the first survey in 2010 and this iteration in 2016–17. Increasing participation remains important, as repeating the survey at European level with regular time intervals can encourage countries to develop their own national surveillance network for LTCFs, as has been the case in the Netherlands, Norway and Sweden, for example [28-30].

One limitation of this survey was its cross-sectional design, as a survey conducted on one single day can be prone to variation. Nevertheless, this methodology was chosen because of its feasibility when applied in settings with limited resources for surveillance and for infection prevention and control, such as LTCFs. Another limitation was that country representativeness was not optimal in all countries and convenience sampling was often used; both of these factors add to the limitations for inter-country comparisons. An additional limitation of our analysis was the large number of LTCFs that did not report any resident with at least one antimicrobial agent on the day of the survey, which may be another consequence of the differences between participating LTCFs and might warrant more sophisticated statistical methods to take this into account in future analyses.

In conclusion, this third PPS provided overall representative data on antimicrobial use in LTCFs across the EU/EEA countries, and demonstrated that continued surveillance for antibiotic use and stewardship practices in LTCFs remains critical. The survey data allow for identifying targets for future antimicrobial stewardship interventions, specifically in LTCFs; for example focusing on prophylaxis for UTIs, following up on the impact of interventions and, ultimately, contributing to the promotion of prudent use of antimicrobials in LTCFs.

## Notes

**Members of the HALT Study Group:** Luigi Segagni Lusignani (Medical University Vienna, Vienna, Austria; luigi.segagnilusignani@meduniwien.ac.at) Reinhild Strauss (Federal Ministry for Labour, Social Security, Health and Consumer Protection (BMAGSK) Vienna, Austria; Reinhild.strauss@sozialministerium.at) Katrien Latour (Sciensano, Brussels, Belgium; Katrien.Latour@sciensano.be) Béatrice Jans (Sciensano, Brussels, Belgium; beatrice.jans@telenet.be) Dana Hedlová (National Institute of Public Health, Prague, Czech Republic; dana.hedlova@uvn.cz) Vlastimil Jindrák (National Institute of Public Health, Prague, Czech Republic; vlastimil.jindrak@gmail.com) Zrinka Bošnjak (University of Zagreb School of Medicine, University Hospital Centre Zagreb, Zagreb, Croatia; zbosnjak@kbc-zagreb.hr) Ana Budimir (University of Zagreb School of Medicine, University Hospital Centre Zagreb, Zagreb, Croatia; abudimir@kbc-zagreb.hr) Elena Gabriel (Ministry of Health, Nicosia, Cyprus; EGabriel@ns.moh.gov.cy) Christian Stab Jensen (Statens Serum Institut (SSI), Copenhagen, Denmark; csj@ssi.dk) Outi Lyytikäinen (National Institute for Health and Welfare, Helsinki, Finland; outi.lyytikainen@thl.fi) Emmi Sarvikivi (National Institute for Health and Welfare, Helsinki, Finland; emmi.sarvikivi@thl.fi) Anne Savey (CPias Auvergne-Rhône-Alpes, University Hospital (HCL), Lyon, France; anne.savey@chu-lyon.fr) Côme Daniau (National Public Health Agency (Santé publique France), Saint Maurice, France; come.daniau@santepubliquefrance.fr) Nicole Schmidt (Healthcare-associated Infections, Surveillance of Antibiotic Resistance and Consumption, Infectious Disease Epidemiology, Robert Koch-Institute (RKI), Berlin, Germany; schmidtn@rki.de) Claudia Ruscher (Applied Infection Control and Hospital Hygiene; Department of Infectious Diseases, Robert Koch-Institute (RKI); Berlin, Germany; ruscherc@rki.de) Maria-Evangelia Adami (Heraklion University Hospital (PAGNH), Heraklion, Greece; emaria.adami@gmail.com) Symeon H. Panagiotakis (Heraklion University Hospital (PAGNH), Heraklion, Greece; simeongpan@hotmail.com) István Veress (Department of Hospital Hygiene and Epidemiological Surveillance, Budapest, Hungary; istvan.veress@emmi.gov.hu) Karen Burns (Health Protection Surveillance Centre, Beaumont Hospital & Royal College of Surgeons in Ireland, Dublin, Ireland; karen.burns1@hse.ie) Helen Murphy (Health Protection Surveillance Centre, Dublin, Ireland; helen.murphy@hpsc.ie) Carla M. Zotti (Dept. Public Health Sciences and Paediatrics, Turin, Italy; carla.zotti@unito.it) Maria Francesca Furmenti (Dept. Public Health Sciences and Paediatrics, Turin, Italy; mariafrancesca.furmenti@unito.it) Justina Avelytė (Institute of Hygiene (HI), Vilnius, Lithuania; justina.avelyte@hi.lt) Murielle Weydert (Ministère de la Famille, de l’Intégration et à la Grande Région, Luxembourg, Luxembourg; murielle.weydert@fm.etat.lu) Branka Petrovska Basovska (Institute of Public Health of Republic of Macedonia – Skopje, Skopje, the former Yugoslav Republic of Macedonia; branka.petrovska@yahoo.com) Dragan Kochinski (Institute of Public Health of Republic of Macedonia – Skopje, Skopje, the former Yugoslav Republic of Macedonia; dragan.kocinski@gmail.com) Michael A. Borg (Mater Dei Hospital & University of Malta, Msida, Malta; michael.a.borg@gov.mt) Mark Bonanno (St. Vincent de Paul Residence, Luqa, Malta; mark.bonanno@gov.mt) Linda Verhoef (National Institute for Public Health and Environment (RIVM), Bilthoven, The Netherlands; 'l.p.b.verhoef@nvwa.nl') Kati Halonen (National Institute for Public Health and Environment (RIVM), Bilthoven, The Netherlands; kati.halonen@rivm.nl) Hanne-M. Eriksen (Norwegian Institute of Public Health, Oslo, Norway; hmer@fhi.no) Horst Bentele (Norwegian Institute of Public Health, Oslo, Norway; hobe@fhi.no) Jadwiga Wojkowska-Mach (Chair of Microbiology, Faculty of Medicine Jagiellonian University Medical School, Krakow, Poland; mbmach@cyf-kr.edu.pl) Beata Mazińska (National Medicines Institute (NMI), Warsaw, Poland; b.mazinska@nil.gov.pl) Pedro Pacheco (Direção-Geral da Saúde (DGS), Lisbon, Portugal; pedrojorgepacheco@gmail.com) Margarida Valente (Direção-Geral da Saúde (DGS), Lisbon, Portugal; margaridavalente@dgs.min-saude.pt) Ljiljana Markovic-Denic (University of Belgrade, Faculty of Medicine, Belgrade, Serbia; markovic.denic@gmail.com) Gordana Krtinic (General Hospital, Subotica, Serbia; gordana.krtinic@bolnicasubotica.com) Mária Kopilec Garabášová (Regional Public Health Authority in Trenčín, Slovakia; garabasova.majka@yahoo.com) Mária Štefkovičová (Alexander Dubcek University in Trenčín and Regional Public Health Authority in Trenčín, Slovakia; tn.stefkovicova@uvzsr.sk) Enric Limon Caceres (VINCat program, Spain & University of Barcelona, Barcelona, Spain; elimon@iconcologia.net) Maria José Torijano Castillo (Epidemiology Area of the Autonomous Community of Madrid, General Directorate of Public Health, Madrid, Spain; mariajose.torijano@salud.madrid.org) Tomas Söderblom (The Public Health Agency of Sweden, Solna Sweden; tomas.soderblom@folkhalsomyndigheten.se) Jenny Hellman (The Public Health Agency of Sweden, Solna, Sweden; jenny.hellman@folkhalsomyndigheten.se) Muhammad Sartaj (HSC Public Health Agency, Belfast, Northern Ireland, UK; muhammad.sartaj@hscni.net) Tony Crockford (HSC Public Health Agency, Belfast, Northern Ireland UK; tony.crockford@hscni.net) Shona Cairns (National Services Scotland, Health Protection Scotland, Glasgow, Scotland, UK; shona.cairns@nhs.net) Cheryl Gibbons (National Services Scotland, Health Protection Scotland, Glasgow, Scotland, UK; cheryl.gibbons@nhs.net) Wendy Harrison (Public Health Wales, Cardiff, Wales, UK; wendy.harrison2@wales.nhs.uk) Christine Jeffrey (Public Health Wales, Cardiff, Wales, UK; Christine.jeffrey@wales.nhs.uk)

## References

[1] European Union (EU) Eurostat. Eurostat – Population projections 2015 based. Projected old-age dependency ratio. [Accessed: 04 Apr 2018]. Europe: EU Eurostat. Available from: http://ec.europa.eu/eurostat/tgm/table.do?tab=table&init=1&plugin=1&pcode=tps00200&language=en

[2] European Union (EU) Eurostat. Healthcare resource statistics - beds. Europe: EU Eurostat; 2017. Available from: http://ec.europa.eu/eurostat/statistics-explained/index.php/Healthcare_resource_statistics_-_beds

[3] World Health Organisation (WHO). Eurostat, OECD. A System of Health Accounts. OECD Publishing. Geneva: WHO; 2011. Available from: http://www.who.int/health-accounts/methodology/sha2011.pdf

[4] Cotter M Donlon S Roche F Byrne H Fitzpatrick F Healthcare-associated infection in Irish long-term care facilities: results from the First National Prevalence Study. J Hosp Infect. 2012;80(3):212-6. 10.1016/j.jhin.2011.12.010 22305100

[5] Rummukainen ML Mäkelä M Noro A Finne-Soveri H Lyytikäinen O Assessing prevalence of antimicrobial use and infections using the minimal data set in Finnish long-term care facilities. Am J Infect Control. 2013;41(4):e35-7. 10.1016/j.ajic.2012.09.007 23332375

[6] Eilers R Veldman-Ariesen MJ Haenen A van Benthem BH Prevalence and determinants associated with healthcare-associated infections in long-term care facilities (HALT) in the Netherlands, May to June 2010. Euro Surveill. 2012;17(34):20252. 22939212

[7] Heudorf U Boehlcke K Schade M Healthcare-associated infections in long-term care facilities (HALT) in Frankfurt am Main, Germany, January to March 2011. Euro Surveill. 2012;17(35):20256. 22958607

[8] Moro ML Ricchizzi E Morsillo F Marchi M Puro V Zotti CM Infections and antimicrobial resistance in long term care facilities: a national prevalence study. Ann Ig. 2013;25(2):109-18. 23471448 10.7416/ai.2013.1912

[9] Wójkowska-Mach J Gryglewska B Czekaj J Adamski P Grodzicki T Heczko PB Infection control: point prevalence study versus incidence study in Polish long-term care facilities in 2009-2010 in the Małopolska Region. Infection. 2013;41(1):1-8. 10.1007/s15010-012-0351-5 23086684 PMC3566398

[10] Nicolle LE Infection prevention issues in long-term care. Curr Opin Infect Dis. 2014;27(4):363-9. 10.1097/QCO.0000000000000071 24921424

[11] van Buul LW van der Steen JT Veenhuizen RB Achterberg WP Schellevis FG Essink RTGM Antibiotic use and resistance in long term care facilities. J Am Med Dir Assoc. 2012;13(6):568.e1-13. 10.1016/j.jamda.2012.04.004 22575772

[12] van den Dool C Haenen A Leenstra T Wallinga J The role of nursing homes in the spread of antimicrobial resistance over the healthcare network. Infect Control Hosp Epidemiol. 2016;37(7):761-7. 10.1017/ice.2016.59 27052880 PMC4926272

[13] Verhoef L Roukens M de Greeff S Meessen N Natsch S Stobberingh E Carriage of antimicrobial-resistant commensal bacteria in Dutch long-term-care facilities. J Antimicrob Chemother. 2016;71(9):2586-92. 10.1093/jac/dkw183 27246237

[14] Cassone M Mody L Colonization with multidrug-resistant organisms in nursing homes: scope, importance, and management. Curr Geriatr Rep. 2015;4(1):87-95. 10.1007/s13670-015-0120-2 25664233 PMC4317322

[15] van Buul LW Veenhuizen RB Achterberg WP Schellevis FG Essink RTGM de Greeff SC Antibiotic prescribing in Dutch nursing homes: how appropriate is it? J Am Med Dir Assoc. 2015;16(3):229-37. 10.1016/j.jamda.2014.10.003 25458444

[16] European Centre for Disease prevention and Control (ECDC) and European Commission. (EC). EU Guidelines for the prudent use of antimicrobials in human health. Stockholm: ECDC; Jun 2017. Available from: https://ec.europa.eu/health/amr/sites/amr/files/amr_guidelines_prudent_use_en.pdf

[17] European Centre for Disease Prevention and Control (ECDC). Protocol for point prevalence surveys of healthcare-associated infections and antimicrobial use in European long-term care facilities – version 2.1. Stockholm: ECDC; 2016. Available from: https://ecdc.europa.eu/sites/portal/files/media/en/publications/Publications/HALT-3-LTCF-PPS-Protocol-v2.1.pdf

[18] World Health Organization (WHO) Collaborating Centre for Drug Statistics Methodology. Guidelines for ATC classification and DDD assignment 2018. Oslo: WHO; 2017. Available from: https://www.whocc.no/filearchive/publications/guidelines.pdf

[19] European Centre for Disease Prevention and Control (ECDC). Point prevalence survey of healthcare associated infections and antimicrobial use in European long-term care facilities. May–September 2010. Stockholm: ECDC; 2014. Available from: https://ecdc.europa.eu/sites/portal/files/media/en/publications/Publications/healthcare-associated-infections-antimicrobial-consumption-point-prevalence-survey-long-term-care-facilities-2010.pdf

[20] European Centre for Disease Prevention and Control (ECDC). Point prevalence survey of healthcare-associated infections and antimicrobial use in European long-term care facilities. April-May 2013. Stockholm: ECDC; 2014. Available from: https://ecdc.europa.eu/sites/portal/files/media/en/publications/Publications/healthcare-associated-infections-point-prevalence-survey-long-term-care-facilities-2013.pdf

[21] McClean P Hughes C Tunney M Goossens H Jans B Jans B European Surveillance of Antimicrobial Consumption (ESAC) Nursing Home Project Group Antimicrobial prescribing in European nursing homes. J Antimicrob Chemother. 2011;66(7):1609-16. 10.1093/jac/dkr183 21596722

[22] Moro ML Jans B Cookson B Fabry J The burden of healthcare‐associated infections in European long‐term care facilities. Infect Control Hosp Epidemiol. 2010;31(S1) Suppl 1;S59-62. 10.1086/655989 20929373

[23] Daneman N Gruneir A Bronskill SE Newman A Fischer HD Rochon PA Prolonged antibiotic treatment in long-term care: role of the prescriber. JAMA Intern Med. 2013;173(8):673-82. 10.1001/jamainternmed.2013.3029 23552741

[24] Ahmed H Davies F Francis N Farewell D Butler C Paranjothy S Long-term antibiotics for prevention of recurrent urinary tract infection in older adults: systematic review and meta-analysis of randomised trials. BMJ Open. 2017;7(5):e015233. 10.1136/bmjopen-2016-015233 28554926 PMC5729980

[25] Lo TS Hammer KD Zegarra M Cho WC Methenamine: a forgotten drug for preventing recurrent urinary tract infection in a multidrug resistance era. Expert Rev Anti Infect Ther. 2014;12(5):549-54. 10.1586/14787210.2014.904202 24689705

[26] Lee BS Bhuta T Simpson JM Craig JC Methenamine hippurate for preventing urinary tract infections. Cochrane Database Syst Rev. 2012;10(11):CD003265. 23076896 10.1002/14651858.CD003265.pub3 PMC7144741

[27] Falcone M, Paul M, Yahav D, Orlando G, Tiseo G, Prendki V, et al. Antimicrobial consumption and impact of antimicrobial stewardship programmes in long-term care facilities. Clin Microbiol Infect. 2018;S1198-743X(18)30559-7. 30076978 10.1016/j.cmi.2018.07.028

[28] Zomer TP VAN DER Maaden T VAN Gageldonk-Lafeber AB DE Greeff SC VAN DER Steen JT Verhoef L Incidence of pneumonia in nursing home residents with dementia in the Netherlands: an estimation based on three differently designed studies. Epidemiol Infect. 2017;145(11):2400-8. 10.1017/S0950268817001339 28669365 PMC9148816

[29] Alberg T, Holen Ø, Salvesen Blix H, Lindbæk M, Bentele H, Eriksen HM. Antibiotic use and infections in nursing homes. Tidsskr Nor Legeforen 2017;137: 357-61. Available from: https://tidsskriftet.no/en/2017/03/original-article/antibiotic-use-and-infections-nursing-homes 10.4045/tidsskr.16.0621 28272565

[30] Public health Agency Sweden (PHAS). Punktprevalensmätning av vårdrelaterade infektioner och antibiotikaanvändning inom särskilt boende i Sverige: Svenska-HALT [Point Prevalence Measurement of Health-Related Infections and Antibiotic Use in Special Accommodation in Sweden: Swedish-HALT]. Stockholm: PHAS; 2017. Swedish. Available from: https://www.folkhalsomyndigheten.se/contentassets/e215ba49156d437381688f4c260cd359/protokoll_svenskahalt.pdf

